# A complexity theory-informed COPC approach to evaluation of mining health programmes

**DOI:** 10.4102/phcfm.v17i1.5056

**Published:** 2025-11-25

**Authors:** Wayne Renkin, Johannes F.M. Hugo

**Affiliations:** 1 Community-Oriented Primary Care Research Unit, Department of Family Medicine, Faculty of Health Sciences, University of Pretoria, Pretoria, South Africa

**Keywords:** community-oriented primary care, complexity theory, transdisciplinary, mining-affected communities, sustainable development goals, programme evaluation, transdisciplinary research, primary healthcare

## Abstract

**Background:**

Conventional evaluation approaches are often designed for predictable, linear systems and fail to capture the non-linear dynamics of complex community health interventions. Health projects in mining-affected communities are typically fragmented, with limited coordination or responsiveness to socio-ecological realities. This study applied complexity theory, community-oriented primary care (COPC) principles, and used a transdisciplinary approach to assess a health and wellness project in such a setting.

**Aim:**

To describe and reflect on the methodology of a complexity-informed assessment of the coherence and contextual alignment of a health and wellness project in mining-affected communities.

**Setting:**

Health and wellness projects funded by the Sishen Iron Ore Company Community Development Trust (SIOC-CDT) and implemented across five municipalities in the Northern Cape and Limpopo provinces, South Africa.

**Methods:**

A complexity-informed, mixed-methods design was used. Data were gathered through document review, field observation, and 12 key informant interviews with implementing agents, health officials and traditional healers. Community-oriented primary care principles and complexity theory guided iterative analysis and were supported by digital tools.

**Results:**

The study identified fragmented implementation, limited household engagement and weak data systems. Despite widespread activity, the lack of integration and adaptive strategy limited systemic effectiveness. However, transdisciplinary engagement, adaptive iteration and co-production of knowledge and reflection facilitated institutional learning and practical proposals for change that are integrated and context sensitive, responding to complexities.

**Conclusion:**

Sustainable health system change in complex settings requires integrated, reflexive and locally grounded approaches that move beyond project-based interventions.

**Contribution:**

This study demonstrated how complexity theory, transdisciplinarity, and community-oriented primary care principles offer a viable methodological framework for adaptive evaluation and systemic learning in community health and development, contributing to the journal’s focus on primary care and community health systems in dynamic contexts.

## Introduction

Mining in sub-Saharan Africa is at the heart of the contemporary global drive for critical mineral extraction.^1^ As such, it has the potential to either improve the economic and social well-being of people in mining communities or become the so-called ‘resource curse’, where the majority of the people in primary producing countries bear the health and social costs of over-exploitation and weak resource governance without enjoying sustained material benefit.^2^ Although jobs are created and economic development takes place, the environmental and social damage can often outweigh the perceived benefits and gains.^3^ These dynamics frequently strain relationships between mining companies and affected communities.^4^

In an effort to mitigate these adverse effects on the large variety of mining-affected communities, mostly in rural poor communities in South Africa, the South African Department of Minerals and Energy, through the *Mineral and Petroleum Resources Development Act*, 2002 (Act No 28 of 2002), mandates the implementation of Social and Labour Plans, which include Mine Community Development Plans. These plans are designed to promote community well-being throughout the operational lifespan of the mine and facilitate sustainability after mine closure.^5^

Following the unbundling of the state-owned company ISCOR Limited in 2001, Kumba Resources Limited (the predecessor of Kumba Iron Ore Limited) and Sishen Iron Ore Company (Pty) Ltd (SIOC) were established.^6^ In 2006, Kumba Iron Ore Limited established the Sishen Iron Ore Community Development Trust (SIOC-CDT) as a vehicle to invest in the long-term development of the communities surrounding its mining activities in the Northern Cape and Limpopo provinces, ensuring communities thrive during and beyond the lifespan of mining operations.^7^ Sishen Iron Ore Community Development Trust holds a 3.06% ownership stake in SIOC, alongside Kumba Iron Ore Limited (75.37%), Exxaro Resources Limited (20.37%) and the SIOC Employee Share Ownership Scheme (1.2%).^8^ The Trust is structured around four key developmental pillars: (1) Education, (2) Health and Wellness (HW), (3) Economic Development and (4) Emergent Needs.^9,10^

This study focuses specifically on the SIOC-CDT’s HW pillar, which is aligned with sustainable development goals (SDGs) 2, 3 and 5 and implemented in partnership with the relevant government Health and Social Development services in the relevant districts. The HW pillar includes 11 active projects (Rea Fola Mobile Clinic; Youth Centre Programme; Comprehensive Eye Care Project; TB and HIV/AIDS Project; Women’s Health; Capacity building for people with Disabilities in Thabazimbi; Upskilling nursing specialities services in the Northern Cape; Comprehensive Maternal and Child Health Project; Substance Use Disorder Interventions; Karabelo: Strengthening Gender-Based Violence and Femicide Response; Ke Botlhokoa: Mental Health Programme), selected on 12 SDG targets and 19 indicators.^7,11,12,13^ The University of Pretoria’s Community-Oriented Primary Care Research Unit (UP COPC RU), Faculty of Health Sciences, was contracted to conduct an assessment of these interventions with a view to understanding their potential impact by 2030.

The social value of this research extends beyond providing accountability for funded interventions; it is aimed at strengthening equity and justice for the populations most affected by mining operations.

Numerous evaluations and studies within health and development settings are inappropriate and are unable to respond to the complexities. They often have limited value beyond a ‘tick-box exercise’. Furthermore, the cycle of proposal, evaluation and presentation often fails to influence implementation in any substantive way.^14,15,16,17^

The scientific purpose of this article is methodological: it presents the design and implementation of the research process used in the assessment, as well as the experience of implementing the methods and the learnings thereof. This article contributes to the knowledge and understanding of using complexity theory-informed research and implementation practices within health and development settings.

This article describes a research approach that integrates principles of community-oriented primary care (COPC) and uses complexity theory-informed and transdisciplinary approaches to support adaptive learning, co-creation of knowledge and context-sensitive programme evaluation.^18,19,20,21,22,23,24^ It focuses not only on assessing project outcomes but also seeking to better understand the complexities of the programmes, context and social realities and to develop responses and interventions that are appropriate and able to respond to the complexities.

We describe the conceptual framework in terms of COPC, complexity theory, and transdisciplinarity. Traditional or conventional research and evaluation approaches and tools are often designed for predictable, structured environments and linear systems where the causalities are straightforward. These methods are often inappropriate or ineffective, as they fail to capture the complexities of a dynamic, unpredictable system, where the causalities are non-linear. Therefore, a complexity theory approach and method are required.^22,23^ The following outlines the conceptual framework of this article by briefly exploring COPC, complexity theory, transdisciplinarity, and the integration of frameworks.

It is widely accepted that to strengthen health systems, a focus on primary healthcare (PHC) is required. Primary healthcare is not just about addressing specific diseases but has to be treated as a system.^21^ Community-oriented primary care, as a systems response and approach to PHC, provides a framework for all healthcare.^25^ Community-oriented primary care is an internationally recognised approach to PHC.^26,27^ It represents an integrated, evidence-based approach to PHC that combines clinical services with public health interventions, ensuring comprehensive, equitable and person-centred care.^21,28^ Community-oriented primary care was developed in South Africa during the 20th century to meet the healthcare needs of poor, underserved and excluded populations and remains an appropriate 21st-century system response to persistent health challenges.^26,28^

Community-oriented primary care mobilises clinical and public health resources in the places where people live and work, designed to enable everyone to contribute to and benefit from health.^25,29^ Community-oriented primary care transforms a fragile, top-heavy health system, where strong, specialised services rest on a weak primary healthcare system and under-resourced households, into a strong, sustainable pyramid by reinforcing primary healthcare and empowering families and community health workers or health promoters at the household level. By integrating health promotion, disease prevention and early intervention into communities at the household level, COPC strengthens the foundation of healthcare, ensuring that households receive proactive and continuous care.

The approach is guided by nine principles: (1) A defined community; (2) analysis of local health needs and assets; (3) prioritisation of health needs and interventions; (4) community participation; (5) a comprehensive approach; (6) an equitable approach; (7) evidence-based and scientific; (8) a multidisciplinary team approach and (9) service integration around users.^21^

There is no single, universally agreed-upon definition of complexity theory. For the purpose of this article, complexity theory is understood as ‘a characteristic of a system’^30^ that ‘conceptualises relationships of components (i.e. individuals) within a system as the foundation from which the properties of a system emerge’.^31^ Complexity theory examines how dynamic interactions among system components give rise to emergent behaviours and properties that cannot be predicted by analysing components in isolation.^32^

The difference between complicated and complex should be noted. A complicated system is an object or system that can be ‘taken apart’ or deconstructed, and each individual part can be studied and understood and be put together again, such as a clock or aeroplane. A complex object or system cannot be understood by taking it apart and examining the individual parts. The object or system is made up of the interaction of the individual parts and emergent properties from the interaction. A complex system must be examined as a whole to be understood and not just the individual parts, such as a cat, socio-ecological or health systems.^30,33,34,35^

A complex system involves dynamic, interconnected components that interact in unpredictable ways, often influenced by feedback loops and emergent behaviours, where the interaction between the agents is more important than the nature of the agents.^36,37^ In a complex system, the causal relationships are non-linear, and outcomes cannot be fully anticipated in advance.^32,36,37^

The study context is within a system that is complex, with multiple interventions and actors (Department of Health, the individuals, organisations, funders, politicians, project implementors, health workers, patients, communities and families), each with their own sets of rules. The engagements, interactions and interventions have a non-linear causality and are unpredictable.

Transdisciplinary approaches purposefully transcend institutional and epistemological boundaries, fostering co-production of contextually relevant knowledge by uniting diverse stakeholders (researchers, practitioners and community members) into collaborative, reflexive inquiry processes.^24,38,39^ Unlike disciplinary or interdisciplinary methods, transdisciplinarity involves:

‘[*A*] reflexive research approach that addresses societal problems by means of interdisciplinary collaboration as well as the collaboration between researchers and extra-scientific actors; its aim is to enable mutual learning processes between science and society.’^39^

There is an imperative of generating actionable, meaningful knowledge that is both scientifically rigorous and practically transformative.^38^ Such research necessitates more than token participation; it involves a deliberate surrender of epistemological sovereignty and the redistribution of power in knowledge creation.

This article integrates complexity theory-informed evaluation with COPC principles and transdisciplinary approaches to support adaptive learning, co-creation of knowledge and context-sensitive programme evaluation.^18,19,20,21,22,23,24^ The approach focuses not only on assessing project outcomes but also on understanding the complexities of programmes, context and social realities and developing responses and interventions that are appropriate and able to respond to the complexities.

The aim of this article is to describe and critically reflect on the methodology of a complexity-informed assessment of the coherence and contextual alignment of the SIOC-CDT HW pillar in mining-affected communities.

The objectives of this article are:

- To evaluate the research process used to assess programme coherence, integration and alignment with the 2030 sustainable development goals (SDGs).
- To analyse how COPC principles and complexity theory-informed data collection, analysis and interpretation.
- To document how transdisciplinary collaboration and reflexive practice shaped the research process.
- To reflect on methodological strengths, challenges and lessons learned for evaluating complex community health interventions.

## Research methods and design

### Study design

The study used a pragmatic, complementary, mixed-method design.^40^ The methodological design was guided by the principles and practices of COPC,^21,28^ and informed and framed by complexity theory,^32,36,37^ supported by transdisciplinarity.^24,38,39,41^ Community-oriented primary care, complexity theory, and transdisciplinarity are elaborated in the discussion section.

A mixed-methods approach,^40^ combining qualitative, quantitative and participatory approaches, enabled the researchers to gain a deeper, more complex understanding of the SIOC-CDT interventions.

This research was situated within a pragmatic paradigm, which prioritises practical relevance and explicitly values intersubjectivity, shared meaning-making and abductive reasoning. It is outcome oriented, prioritising the identification of practical solutions to social problems through knowledge creation and joint action. Pragmatism enables the use of diverse data sources, even where inconsistencies or data gaps are present and encourages methodological adaptability in response to context. It also foregrounds reflective inquiry as an intentional component of the research process – both as a means of learning and a mechanism for integrating researcher experiences, literature and participant engagement. Additionally, pragmatism enhances the transferability of findings by situating them within specific contexts while ensuring their broader applicability, thereby contributing to both disciplinary theory and practice.^40,42^

Throughout the research, the researchers engaged in an iterative process of observation, experience and sense-making, which included ongoing reflection on their evolving understanding of the material, local context and interactions. As a formal methodological component aligned with the pragmatic paradigm, reflective inquiry was deliberately integrated into the study. Researchers documented their positionality, insights and the interpretive dialogue between literature, context and practice. This reflective dimension – also expressed through narrative elements in the article – enabled the team to navigate ambiguity, challenge assumptions and interpret emergent patterns in both data and relationships. It also supported the study’s adaptive logic and transdisciplinary orientation, recognising that knowledge creation was shaped by the researchers’ learning and change over time.

The research design was also interdisciplinary and transdisciplinary. The SIOC-CDT Education pillar commissioned the University of Pretoria’s Centre for Evaluation and Assessment, Faculty of Education, to conduct a similar impact assessment. Interdisciplinary collaboration occurred between the researchers from UP COPC RU and the Centre for Evaluation and Assessment. Transdisciplinary collaboration occurred during the process of co-production of knowledge with the SIOC-CDT staff, project teams and the Department of Health. The specific evaluation framework that guided this complexity-informed, pragmatic approach is detailed below.

This study employed complexity theory as its primary evaluation framework, informed by COPC principles. Complexity theory has emerged as an established and appropriate evaluation framework for health interventions, particularly in dynamic, multi-stakeholder environments where traditional linear evaluation approaches prove inadequate.^22,32,43,44,45,46,47,48,49,50^ As Greenhalgh and Papoutsi argue, complexity-informed evaluation is essential for ‘studying interventions that work differently in different contexts’ and for understanding how ‘dynamic interactions among system components give rise to emergent behaviours’.^32^

The complexity-informed evaluation framework recognises that health interventions in mining-affected communities operate within complex adaptive systems characterised by non-linear causality, emergent properties and unpredictable interactions between multiple agents.^22,46,47^ Unlike conventional evaluation approaches that seek to isolate variables and establish linear cause-and-effect relationships, complexity theory evaluation embraces uncertainty and focuses on understanding system interactions, adaptation processes and emergent outcomes.^46,48^ This framework is particularly suited to evaluating multi-component interventions where *the whole is greater than the sum of its parts* and where context significantly influences implementation and outcomes.^43,44^

Within this complexity-informed framework, COPC principles served as both analytical lens and evaluative criteria. The nine COPC principles^21^ provided a structured approach to assess how well the HW interventions aligned with COPC best practices, while complexity theory guided the methodological approach to capture system-level interactions, emergent properties and adaptive responses. This dual framework enabled the evaluation to move beyond simple outcome measurement toward understanding the systemic coherence, contextual alignment and adaptive capacity of the HW programme within the broader socio-ecological context of mining-affected communities.

While sharing some similarities with realist evaluation, notably the concern with context and mechanisms,^51^ this study differs in its epistemological orientation. Realist evaluation is explicitly theory driven, seeking to test middle-range theories of how programmes work in specific contexts.^52^ By contrast, this approach was complexity informed: it emphasised non-linearity, emergence and adaptive dynamics within complex adaptive systems.^32,50^ Rather than testing predefined hypotheses, the evaluation focused on reflexive inquiry, relational engagement and iterative adaptation as methodological commitments.

The researchers used a pragmatic approach, which emphasises intersubjectivity as a way to move beyond the artificial separation between subjective and objective perspectives. The pragmatic approach argues against the forced dichotomy between subjective and objective views. The researchers engaged with the participants, as well as the SIOC-CDT team, to achieve a sufficient degree of mutual understanding, and the researchers moved back and forth between various frames of reference and engaged with the literature.^18,23^ In the context of limited or incomplete knowledge about cause and effect, implementation processes must tie analytical and management efforts to explicit questions as to how change happens in their context.^9^ As it cannot be taken for granted how change will happen, it is very important to make explicit ideas and assumptions underlying implementation and to test and reflect on this purposefully.^22^

A basic set of assumptions framed the research process:

- Complexity:
  ■ The context and environment of the interventions are complex.
  ■ The interactions between the agents are more important than the agents themselves.
  ■ The different projects, interventions, organisations, funders, implementing organisations and the stakeholders each have their own rules of engagement/functioning.
  ■ The cause and effect are non-linear.
  ■ There is a great deal of uncertainty.
- Collaboration:
  ■ Siloed interventions are ineffective and insufficient for long-term impact.
  ■ Collaboration, integration and co-design are essential.
  ■ Active participation enhances the evaluation and implementation processes.
- Primary health care:
  ■ A strong healthcare system is built on strong PHC foundations.
  ■ Health begins at home; therefore, the household is key to health interventions and actions.
  ■ COPC is the most contextually appropriate model for strengthening PHC.
- Data:
  ■ Available data are incomplete, inappropriate or inconsistent.
  ■ Documentation processes are not standardised or rigorously applied.
  ■ Process and decisions are not rigorously documented.

### Setting

SIOC-CDT’s mandate is to implement projects and interventions across five municipalities in two provinces, Limpopo and Northern Cape, in South Africa.^9^ The study setting was within the areas where SIOC-CDT implements its projects. The municipalities are:

- Gamagara Local Municipality, John Taolo Gaetsewe District, Northern Cape province.
- Ga-Segonyana Local Municipality, John Taolo Gaetsewe District, Northern Cape province.
- Joe Morolong Local Municipality, John Taolo Gaetsewe District, Northern Cape province.
- Tsantsabane Local Municipality, ZF Mgcawu District, Northern Cape province.
- Thabazimbi Local Municipality, Waterberg District, Limpopo province.

### Study population and sampling strategy

The research used purposive sampling to identify key stakeholders involved or connected to SIOC-CDT’s HW interventions. Data sources included:

- Organisational reports and internal documents, both publicly available and provided by SIOC-CDT.
- Key informant interviews with implementing agents funded by SIOC-CDT.
- Interviews with Department of Health representatives.
- Key informant interviews with other relevant stakeholders.

Implementing organisations were selected as they are funded by SIOC-CDT to implement projects. Department of Health representatives were selected based on the existing relationships with SIOC-CDT. Contact details were facilitated by the SIOC-CDT HW team.

### Data collection

In keeping with the pragmatic and complementary methodological approach, the study drew on documentary, observational and qualitative data sources:

- Desktop review of SIOC-CDT strategy documents, internal and project reports, project proposals and the 2022 HW rapid assessment.^53^
- Review of relevant published literature.
- Attendance at review workshops and team meetings with SIOC-CDT staff.
- Twelve in-depth, semi-structured key informant interviews with implementing agents and external stakeholders (Department of Health and traditional healers).
- Observational notes compiled by the researchers.

The desktop review included the following types of documents:

- Strategy documents.
- Reports.
- Project proposals.
- A 2022 HW rapid assessment report.^53^

These documents were analysed using COPC principles.^21^

A total of 12 semi-structured, open-ended interviews were held with eight implementing organisations representing nine SIOC-CDT projects (virtual interviews), and three external stakeholders (one traditional healer interview – in-person; two Provincial Department of Health interviews – one health area manager; one District Director, Strategic Planner Officer, District Director Support – virtual). The external stakeholders included a traditional healer and the Department of Health in the Tsantsabane sub-district and the John Taolo Gaetsewe (JTG) district. The interviews took place from 03 December 2024 to 23 January 2025.

All interviews were audio-recorded with verbal consent. The reviewers transcribed all the recordings using TurboScribe.ai. One researcher conducted transcription quality control. The interviews were semi-structured and open ended. The interview started off with the researchers giving an overview of the impact assessment being conducted, ongoing findings and learnings, proposed models for improving collaboration, integration and community-oriented healthcare, as well as a strong focus on the ‘home’. Participants were invited to reflect on:

- Their view of the proposed approach.
- Their potential contribution to implementation.
- The support they would require.

Interviewers probed emerging probed emerging themes based on participant responses.

### Data analysis

Data analysis used both deductive and inductive approaches:

- SIOC-CDT documents were reviewed and analysed using a COPC-informed matrix.
- Key informant interviews were analysed. Transcriptions were processed using Google NotebookLM (https://notebooklm.google.com/), an AI-assisted tool that supported abductive reasoning, aligned with the pragmatic paradigm.
- Observational notes and reflections were triangulated with interview and document data.
- Results and learnings were shared with participants for feedback and further knowledge creation.

The use of both human and machine-assisted thematic analysis enhanced the transparency and rigour of the qualitative findings while allowing flexibility to accommodate contextual complexity. Table 1 outlines the objectives with the relevant methods, data sources, and the relevant analytical lens.

**TABLE 1:** Summary: Objectives, methods, data sources, analytical lens.

Objective	Methods	Data sources	Analytical lens
Evaluate research process and programme coherence	Document review	SIOC-CDT strategy documents, project reports, proposals	COPC matrix analysis
Analyse COPC and complexity theory application	Key informant interviews (*N* = 12)	Implementing agents, Department of Health officials, traditional healers	Complexity-informed thematic analysis
Document transdisciplinary collaboration	Participatory workshops and observation	SIOC-CDT staff, Board, community stakeholders	Transdisciplinarity framework
Reflect on methodological lessons	Reflexive inquiry, iterative analysis	Research team field notes, synthesis discussion	Pragmatic paradigm

### Ethical considerations

Ethical clearance to conduct this study was obtained from the University of Pretoria’s Faculty of Health Sciences’ Research Ethics Committee (No. 459/2024).

## Results

This section presents a thematic and chronological account of the research process undertaken during the impact assessment of the SIOC-CDT HW pillar. In alignment with the article’s methodological focus, the emphasis is on *how* the research was implemented in practice, and how the process evolved in response to complexity, context, and disruption. Rather than presenting findings, this section outlines the phases of engagement, reflection, adaptation and co-learning that shaped the research approach.

### Establishing the evaluation approach

The research process commenced with initial engagements between the UP COPC RU research team and SIOC-CDT. These early interactions were marked by notable discomfort, as stakeholders grappled with differing expectations regarding the scope, purpose and nature of the assessment. Reflective meetings held in October 2024 surfaced significant misalignments, particularly concerning the intended outcomes of the evaluation.

SIOC-CDT sought clear assessments of the effectiveness and impact of individual projects. However, the research team contended that such evaluations were not feasible without household-level data and broader contextual insights. The fragmented nature of existing data and the absence of a community-based perspective further constrained the potential for isolated impact assessments.

These misalignments reflected deeper divergences in purpose, effort and orientation. Yet, they also became a critical point of entry into the complexity of the system. Rather than imposing a predefined methodology, the research team adopted an adaptive stance – responsive to evolving stakeholder dynamics and contextual realities.

A desktop review of programme documentation, conducted between July 2024 and October 2024, provided an initial understanding of the HW pillar and all its projects. The draft report produced during this phase laid the groundwork for subsequent inquiry. Concurrently, quarterly feedback meetings of all the HW projects in Thabazimbi and Kathu in August 2024 offered vital opportunities for clarification, trust-building and mutual orientation.

### Adapting to disruption and data limitations

As the research progressed, significant challenges emerged around the availability and quality of programme data. It became clear that there were limits and deficiencies in the data. These constraints necessitated a methodological approach that is responsive, adaptive and flexible with greater emphasis on participation and joint knowledge creation. The research design was adapted to foreground reflective inquiry, stakeholder engagement and contextual understanding.

Semi-structured interviews were conducted between November 2024 and January 2025, offering deeper insight into the experiences and perspectives of key actors. The use of digital tools such as NotebookLM and turboscribe.ai supported data transcription, synthesis and iterative analysis.

### Collaboration through participation and practical engagement

The research process assumed that collaboration is essential. Collaboration develops through purpose, respect, effort and safety. Based on that, the research and project teams engaged until functional collaboration was achieved.

From December 2024 to January 2025, the team co-developed a practical proposal for change, informed by the emergent themes and guided by COPC principles. This proposal aimed to support future systems strengthening by addressing gaps and opportunities in the HW pillar, identified through the research process. This proposal was not accepted by the Board, which led to further engagements about integration and collaboration. This required the proposal to change from a focus only on HW to an integrated, transdisciplinary approach and research team that integrates all four pillars of SIOC-CDT.

Two stakeholder workshops were convened in January 2025 – one focusing on education and the other on HW. These workshops created dialogical spaces for reflection, joint sensemaking and strategic prioritisation. They also fostered interdisciplinary engagement, enabling cross-sectoral insights and collaborative exploration of feasible interventions.

The executive summary of the assessment was circulated in draft form in January 2025 and finalised in February 2025, ensuring stakeholder feedback was incorporated. These outputs facilitated ongoing dialogue and validation among internal teams, external partners and community representatives.

### Reporting, dissemination and dialogue

The draft report was submitted in October 2024, capturing the early synthesis of insights. A culminating symposium was held in November 2024, bringing together diverse stakeholders to engage critically with the emerging findings and share reflections. This event helped shape the framing of subsequent engagements.

Following the workshops and development of the practical proposal, the final report was completed in February 2025.^54^ This was followed by presentations to the SIOC-CDT Executive Committee and Board in February and March 2025, respectively. These sessions were not limited to dissemination; rather, they were designed to stimulate institutional reflection and to consider implications for strategic planning, decisions and resource allocations.

### Reflections on complexity, reflexivity and learning

The implementation of this research project offered rich insights into the practice of complexity-informed evaluation. Key moments of disruption, such as data gaps and institutional tension, became opportunities for methodological learning. Reflexivity was not a discrete phase but a continuous orientation that informed the research team’s choices, adaptations and relationships.

Importantly, the chronology of events was constructed retrospectively, as the research process itself did not unfold linearly. The research team had to remain responsive and flexible, constantly adapting to contextual realities and emergent needs. This adaptability was crucial for maintaining relevance and legitimacy in a dynamic and multi-stakeholder environment.

The process also highlighted the importance of positionality, joint knowledge creation, and embedded engagement. Rather than treating evaluation as a linear or objective exercise, the team adopted a pragmatic stance grounded in complexity theory and COPC principles. The research evolved in real time, shaped by ongoing interaction with stakeholders, emergent questions and systemic constraints.

The research process itself served as an expression of complexity-aware practice, namely reflexive, relational and attuned to context. The methodological implications of this approach are explored further in the discussion.

## Discussion

### Key findings

This study examined the methodological processes of evaluating SIOC-CDT’s HW interventions in mining-affected communities through the combined lenses of COPC, complexity theory and transdisciplinarity. The evaluation process not only evaluated projects individually but also the interactions between the projects and stakeholders. The discussion reflects on how these frameworks shaped and were shaped by the research process. Rather than focusing on programme outcomes, the analysis highlights how these frameworks informed the evaluation process and how the research evolved in response to contextual dynamics.

### Discussion of key findings

#### Community-oriented primary care as an analytical lens and framework

**FIGURE 1:**
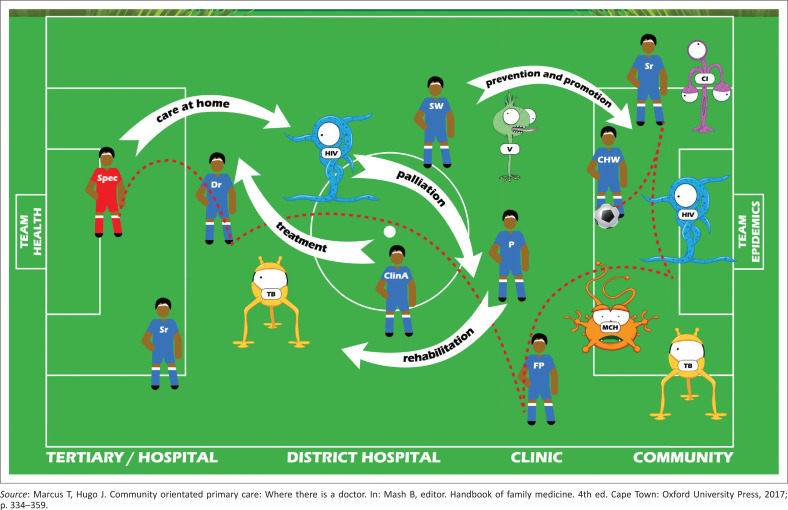
Community-oriented primary care: Changing the game of primary health.

Community-oriented primary care principles were applied as an analytical lens, as well as a way of communicating the results. As an analytical lens, it revealed systemic weaknesses in SIOC-CDT’s HW programmes, particularly the absence of defined populations, limited household-level engagement and fragmented service delivery. This was illustrated and communicated through the metaphor of a soccer game (Figure 1)^25^, to move ‘away from defensive play in your own goal posts’ (care within institutions), to be offensive and scoring ‘goals against disease in the places where people live and work – the home, the factory and the farm’.^25^

The deficiencies and systemic weaknesses of the HW programmes contrasted sharply with COPC’s emphasis on community definition, participatory approaches, care at the household level and integrated care.^21,25,27,28,29^ This framework enabled stakeholders to reframe evaluation from a narrow focus on discrete project outcomes towards broader questions of systemic coherence, accountability and sustainability while simultaneously providing a shared language during workshops and reporting that made complex evaluation findings accessible to diverse stakeholders.

#### Complexity in practice

The environment and systems of the HW projects are complex. The evaluation demonstrated how health interventions unfold within complex adaptive systems characterised by non-linearity, emergence and unpredictability.^32^ Rather than seeking simplified causal explanations and determining linear cause and effect, the research team embraced complexity as a condition of practice. Misaligned expectations between SIOC-CDT and the research team, compounded by poor data quality, necessitated adaptive reframing of aims and methods. The iterative development and ongoing engagements exemplified emergence: new directions arose from interaction and negotiation rather than from a predefined plan. Complexity theory thus provided both an explanatory frame and a practical guide for methodological flexibility. As Greenhalgh and Papoutsi^32^ argue, complexity-informed research must be able to ‘handle the unknown, the uncertain, the unpredictable, and the emergent’ by engaging in collaborative, reflexive and adaptive learning processes.

#### Reflexivity, collaboration and transdisciplinarity

In complex systems, collaboration is not merely a technical function or coordination but a relational practice. The quality of collaboration is characterised by mutual respect, safety, shared vulnerability, joint knowledge creation, and shared purpose.^55^ Reflexive practice was central throughout, turning initial discomfort and misalignment into opportunities for methodological learning. Shared vulnerability and trust-building allowed stakeholders to move from defensive posturing to co-creation and joint problem-solving, aligning with calls for genuine transdisciplinary research.^38,39^ Throughout the research process, the research team intentionally fostered a culture of non-judgemental interaction, where critical feedback could be offered and received without blame or defensiveness. By recognising the influence of power and perspective, the evaluation created dialogical spaces that enabled diverse actors to contribute to joint knowledge creation. This aligns with Daniel Coyle’s^55^ emphasis on building cultures of safety, effort and purpose as preconditions for productive group dynamics.

Collaboration became not only a methodological requirement but an ethical commitment. It also became both the medium and message: this kind of practice is required to respond to complex challenges and community health.

#### Emergence: Iterative learning and proposal development

In line with complexity theory, understanding did not precede action; it emerged through iterative engagement, feedback loops, and adaptive reframing. Each stage of the research process, desktop reviews, interviews, stakeholder workshops, draft reports and proposal development, served as a mechanism for sensemaking. Proposals were not fixed outputs but tools for reflection, inviting critique, and enabling transformation. For example, an early health-focused project proposal, though not accepted by the Board, catalysed a shift towards a broader, transdisciplinary integration across all four of SIOC-CDT’s development pillars.

This iterative dynamic illustrates the concept of emergence, whereby patterns, insights and plans evolved through cycles of action and reflection. Crucially, these iterations were not linear. They reflected the complex and adaptive nature of the system, where the introduction of new information or relationships altered the system’s direction. The workshops exemplified how emergent insights could be translated into proposals and strategies that responded to evolving understandings of context, need and feasibility.

Thus, the assessment did not seek definitive answers but fostered ongoing inquiry. The process embraced uncertainty and used it as a resource for innovation, consistent with calls in the literature for methodologies that can ‘handle the unknown, the uncertain, the unpredictable and the emergent’.^32^

#### Working with uncertainty and data limitations

Uncertainty is a key characteristic of a complex system and therefore unavoidable. It is impossible to predict the outcomes of the future state of a complex system.^35^ This is especially true of health settings. Complexity theory offers a way and framework to deal with uncertainty.^34^

Complex problems are characterised by change processes that are not amenable to detailed forecasting or to anticipating all potential outcomes. Interventions and programmes in complex environments often must be implemented with limited knowledge and a robust understanding of cause and effect. The most appropriate interventions to address the problem might not be well understood. Therefore, there is a need for adaptation given the inherent uncertainties. Success depends on assessing and adapting to emerging signals and changing situations. The processes of the interventions should be designed to facilitate learning and adaptation. Strategy and planning remain relevant, but it is crucial to acknowledge levels of uncertainty and ambiguity. Traditional tools are ineffective in a complex environment, as they are based on inappropriate assumptions. In complex environments, greater emphasis should be placed on learning and observing the emergent during implementation and building flexible and adaptive capacity.^22^

In the assessment, uncertainty was compounded by the quality and availability of data, fragmented and inconsistent programme data, variability of stakeholders and the contexts. Rather than viewing uncertainty as a constraint, the data uncertainty was turned into a resource and a condition to work with. The uncertainty foregrounded learning, reflection and relational engagements.

#### Knowledge translation as practice

In complex systems, knowledge translation is not a discrete post-research task but an embedded, continuous practice. In this study, knowledge was not disseminated from researcher to practitioner, but co-produced through participatory engagement, situated dialogue and transdisciplinary collaboration. Practical outputs – such as the development of integrated proposals and the articulation of household-focused models – were both products and vehicles of knowledge translation.

In a complex system, where knowledge is fluid and dynamic, knowledge will contain uncertainty.^35^ Therefore, complexity theory-informed research enables a broader epistemological shift: from linear knowledge transfer to iterative co-construction.

The researchers’ positionality within the system, their ethical commitment to shared learning and their methodological openness to emergent insight all contributed to a relational mode of knowledge translation. The knowledge generated was thus both contextually grounded and institutionally relevant, culminating in strategic documents and interactions that stimulated reflection, adaptation and planning across multiple levels of SIOC-CDT’s operations.

Transdisciplinary engagement further amplified this practice. As noted by Jahn et al.^39^ and Mitchell et al.,^38^ transdisciplinarity requires the surrender of epistemological sovereignty and the creation of mutual learning spaces. The study’s integration of health, education and enterprise perspectives, alongside community and institutional voices, exemplifies this approach. By embedding knowledge translation within the research process itself, the project contributed to organisational learning and adaptive capacity, offering a model for complexity-responsive evaluation in similarly dynamic contexts.

#### Transdisciplinary, positionality and power

In the context of this assessment, transdisciplinarity was adopted not only as a methodological choice but as an epistemic and ethical stance. It required the research team and institutional actors to interrogate their positionality, critically examining embedded power dynamics and embracing mutual learning. This orientation moved beyond extractive research models, valuing diverse epistemologies, value systems and lived experiences as integral to addressing social-ecological complexity.

The engagement process integrated perspectives from health and education, alongside the insights of SIOC-CDT staff, implementing partners, external stakeholders, and community members. Positionality was approached reflexively throughout, creating dialogical spaces for collaborative learning and systemic insight. In embracing complexity, non-linearity and emergence, the transdisciplinary stance proved instrumental in navigating institutional tensions and co-producing knowledge that was both relevant and relational.

### Implications for methodological innovation

The study demonstrates that evaluating complex community health interventions requires a shift from linear measurement towards adaptive learning systems. Integrating COPC and complexity theory enabled the evaluation to foreground systemic coherence, participatory knowledge production and household-level engagement as critical determinants of impact. The methodological novelty lies not only in the use of complexity-informed frameworks but also in the reflexive, transdisciplinary practice that generated relational shifts within institutional systems. Such approaches may offer a viable model for evaluating and strengthening health programmes in other dynamic and uncertain contexts.

### Strengths and limitations

The study explores the use of COPC principles, guided by complexity theory, to assess health programmes. It offers a contextual approach to not only assessment but also knowledge creation and the development of solutions. Combined with transdisciplinary engagement and participatory reflection, it supports collaborative learning.

The pragmatic, mixed-methods design enables the research process to be responsive in a dynamic environment.

The embeddedness of the research team, their observations, with the evolving nature of the programmes and communities, as well as the disruptions that necessitated adaptation, may have limited replicability. This article does not provide a blueprint for assessments and research within complex environments, but it offers reflections about the researchers’ experience and the implementation of the project. These limitations were addressed by reflecting on the process within different contexts, which should guide other researchers to learn from this process and adapt it in their particular context.

### Implications

As the environment of health and development work is complex, researchers and practitioners should work with the complexity and be able to function within the uncertainty. Researchers should use complexity-informed research methodologies that can capture the complexity, as opposed to traditional research methods that rely on linear approaches. Reflexive practice, positional awareness, and collaborative sensemaking should be central components of both design and implementation. Institutional stakeholders should adopt transdisciplinary, and complexity-informed approaches to address social determinants of health across sectors.

Funders and organisations within health and development work should shift away from a traditional understanding of linear cause and effect, implementing and funding siloed projects and programmes. Collaborative projects and programmes should not only be encouraged but prioritised. Efforts should prioritise household-level engagement and integrated service delivery to achieve systemic change.

## Conclusion

The assessment of SIOC-CDT’s HW pillar revealed significant effort and activity. However, the lack of integration between projects, inconsistent collaboration among stakeholders, limited quality data and limited engagement at the household level critically constrained the potential for long-term systemic impact. While numerous interventions were implemented, their ability to collectively advance the vision of thriving communities, achieving their 2030 Vision, which is aligned with SDGs 2, 3 and 5, remained limited.

This study highlights that HW interventions within mining-affected communities must go beyond fragmented project delivery. Instead, they require coherent, integrated systems grounded in COPC, community participation and adaptive learning. Using the principles of COPC and complexity theory, this research provided a methodological and epistemological framework for assessing health interventions within dynamic and uncertain environments.

The research process itself – characterised by reflexivity, adaptive iteration and transdisciplinary engagement – demonstrated how complexity-informed methodologies can generate not only insights but also relational shifts within institutional systems. By working with uncertainty and embracing stakeholder dialogue, the research team co-produced knowledge that was both contextually relevant and strategically actionable.

The findings of this article do not provide definitive answers but instead lay the groundwork for an evolving, integrative response to complex challenges. The proposed shift from siloed interventions to integrated, household-centred approaches marks a fundamental realignment in programme design, implementation and evaluation. A subsequent article will explore the findings and recommendations that emerged from this complexity-informed assessment.
